# Development of a Pan-*Filoviridae* SYBR Green qPCR Assay for Biosurveillance Studies in Bats

**DOI:** 10.3390/v15040987

**Published:** 2023-04-17

**Authors:** Jessica Coertse, Marinda Mortlock, Antoinette Grobbelaar, Naazneen Moolla, Wanda Markotter, Jacqueline Weyer

**Affiliations:** 1 Centre for Emerging Zoonotic and Parasitic Diseases, National Institute for Communicable Diseases, A Division of the National Health Laboratory Service, Johannesburg 2131, South Africa; 2 Centre for Viral Zoonoses, Department of Medical Virology, Faculty of Health Sciences, University of Pretoria, Pretoria 0001, South Africa; 3 Department of Microbiology and Infectious Diseases, School of Pathology, University of Witwatersrand, Johannesburg 2131, South Africa

**Keywords:** pan-filovirus, surveillance, bats, SYBR Green, qPCR

## Abstract

Recent studies have indicated that bats are hosts to diverse filoviruses. Currently, no pan-filovirus molecular assays are available that have been evaluated for the detection of all mammalian filoviruses. In this study, a two-step pan-filovirus SYBR Green real-time PCR assay targeting the nucleoprotein gene was developed for filovirus surveillance in bats. Synthetic constructs were designed as representatives of nine filovirus species and used to evaluate the assay. This assay detected all synthetic constructs included with an analytical sensitivity of 3–31.7 copies/reaction and was evaluated against the field collected samples. The assay’s performance was similar to a previously published probe based assay for detecting Ebola- and Marburgvirus. The developed pan-filovirus SYBR Green assay will allow for more affordable and sensitive detection of mammalian filoviruses in bat samples.

## 1. Introduction

In accordance with the 2021 taxonomic report of the International Committee on the Taxonomy of Viruses, *Filoviridae* includes six genera [1]. The *Cuevavirus*, *Dianlovirus*, *Striavirus,* and *Thamnovirus* genera each contain a single species, *Lloviu cuevavirus*, *Mengla dianlovirus*, *Xilang striavirus*, and *Huangjiao thamnovirus*. Each of these species have a single virus member, Lloviu virus (LLOV), Měnglà virus (MLAV), Xilăng virus (XILV), and Huángjiāo virus (HUJV), respectively. The genus *Ebolavirus* contains six species, *Bombali ebolavirus*, *Bundibugyo ebolavirus*, *Reston ebolavirus*, *Sudan ebolavirus*, *Tai Forest ebolavirus*, and *Zaire ebolavirus*, each with a single virus member, Bombali virus (BOMV), Bundibugyo virus (BDBV), Reston virus (RESTV), Sudan virus (SUDV), Taï Forest virus (TAFV) and Ebola virus (EBOV), respectively. The genus *Marburgvirus* contains a single species, *Marburg marburgvirus*, with two virus members, Marburg virus (MARV) and Ravn virus (RAVV) [1]. All filoviruses are associated with mammalian hosts except XILV and HUJV, which have been reported from actinopterygian fish in Asia [2].

Mammalian-associated filoviruses can cause life-threatening viral hemorrhagic fever in humans and non-human primates [3]. The first filovirus-associated hemorrhagic fever outbreak was reported in 1967 in laboratory workers in Germany and Serbia [4]. In the 55 years since this first description, more than 40 filovirus outbreaks have been reported [5], with the most significant occurring from 2014 to 2016 in West Africa involving EBOV. This outbreak primarily affected three West African countries with cross-border spread in Sierra Leone, Liberia, and Guinea (i.e., across border spread). Nearly 30,000 cases were diagnosed during this epidemic and more than 11,000 deaths were recorded [6]. Although the exact zoonotic transmission mechanism for filoviruses remains to be fully understood, animal-to-human infections appear to be rare, but after the initial introduction, the subsequent human-to-human transmission through close contact is efficient [3].

Despite the significant impacts of outbreaks and intense research efforts, the natural reservoirs for filoviruses have remained elusive for decades. Certain species of bat are implicated as natural hosts for some filoviruses. The experimental infection of insectivorous and fruit bats supported the replication of EBOV, resulting in high viral titers without developing disease, suggesting bats as potential reservoirs [7]. This hypothesis was supported by the detection of EBOV RNA and antibodies in three bats species (i.e., *Epomops franqueti*, *Hyspignathus monstrosus,* and *Myoncteris torqata*) [8]. Shortly thereafter, MARV and RAVV RNA were also detected in bats, *Miniopterus inflatus*, *Rhinolophus eloquens*, and *Rousettus aegyptiacus,* followed by viral isolation [9,10]. Numerous isolates have been made from *R*. *aegyptiacus* bats over several months, indicating that colonies can harbor MARV over extended periods of time [10]. More recently, LLOV was detected in *Miniopterus* sp. bats in Spain [11] and Hungary [12], with successful virus isolation from a bat blood sample [13].

Using a broadly reactive nested filovirus polymerase chain reaction (PCR) assay as a detection tool, BOMV was identified in two bat species in Sierra Leone [14] and in Kenya and Mozambique using a two-well pan-filovirus reverse transcription real-time PCR (RT-qPCR) assay targeting the RNA-dependent RNA polymerase (L) gene [15,16]. A broadly reactive, nested filovirus assay targeting the L-gene was also used to identify MLAV in bat species in China [17,18]. A human apathogenic filovirus, RESTV, was isolated in 1989 in macaques [19] and has subsequently been shown to infect various animal species including bats in the Philippines [20]. Several hemorrhagic fever outbreaks caused by BDBV and SUDV have been reported in humans [21], while TAFV has only been identified in a single non-fatal human infection [22]. However, no known bat hosts are currently associated with BDBV, SUDV, and TAFV. Although several assays have been published for the detection of filovirus RNA and filovirus commercial assays are becoming increasingly available, these assays have been developed for the detection of filoviruses associated with outbreaks in humans (to date) (i.e., the *Ebolavirus* and *Marburgvirus* genera), with multiple primers and probes per assay (reviewed in [23]). In 2019, Jääskeläinen and co-workers described using a pan-filovirus assay that included two reactions with multiple primers and probes for the detection of the L-gene of members of the *Ebolavirus* and *Marburgvirus* genera. Although this assay was shown to be sensitive (limit of detection of 9.4–1151 copies per reaction) and specific (100%) for the filoviruses tested, it was noted that in silico analysis of the primer and probe sets indicated several mismatches with other bat-related filoviruses [24].

In addition to filoviruses, bats are hosts to a large diversity of zoonotic (and possibly zoonotic) viruses, and as a result, viral discovery and host surveillance studies have increased [25]. While pathogen discovery in bats is becoming more widespread, only a small proportion of the more than 1400 bat species have been targeted for viral discovery [21,26]. Although pathogen surveillance in bats is important from an animal and human health perspective, considering that we have not saturated the viral discovery curve for most bat species [21], it is imperative that the crucial role of bats in ecosystems is not overlooked [27]. Additionally, studies have shown that non-lethal sampling does not decrease the chances of viral detection and that testing more bat species for a broader number of viral families would be efficient for virus discovery [25]. Non-lethal sampling limits the amount of biological material that can be safely collected from a bat, the smallest bat weighing only 2 g with a median of approximately 14 g [28], necessitating the need to optimize pathogen testing strategies. Here, we report on the development of a two-step SYBR Green qPCR assay targeting a region of the nucleoprotein (NP) gene for the detection of mammalian filoviruses. Utilizing a random priming strategy for complementary DNA (cDNA) synthesis allows for multiple assays to be performed from a single preparation, permitting wider pathogen surveillance. In contrast to previous studies that require multiple reactions or multiple assays (pan-filovirus and genus specific assays targeting the L- and NP genes) [14,18,24], the SYBR Green assay will only require a single reaction to detect all currently described filoviruses. This approach is more economical, efficient, and allows for broader filovirus surveillance when using limited sample material.

## 2. Materials and Methods

### 2.1. Primer Design

The ClustalW subroutine of BioEdit Sequence Alignment Editor version 7 [29] was used to create a multiple alignment of representative sequences for the mammalian filoviruses and unclassified bat-related filoviruses (Table S1) available on Genbank (https://www.ncbi.nlm.nih.gov/, accessed on 12 November 2020). A total of 115 nucleoprotein (NP) sequences were used to identify regions of homology for primer design. A single degenerate primer set was designed, Filo For: 5′-GRGARTAYGCICCITTYGC-3′ (binding position 1340 on MG572235) and Filo Rev: 5′-AGYTGYTGRTAYTGYTCICC-3′ (binding position 1477 on MG572235).

### 2.2. Preparation of Standard Templates

**Figure 1:**
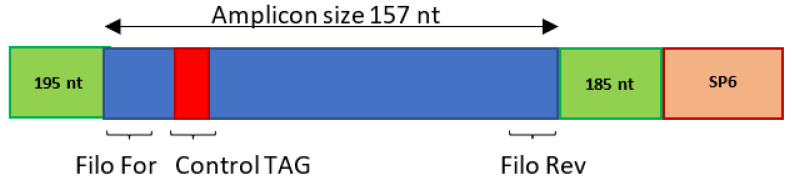
Graphic representation of the synthetic constructs designed for use as assay controls. Each construct consists of a 537-nucleotide partial nucleoprotein sequence of the selected viral species (Table 1). The expected amplicon size for the filovirus products was 157 bp. The control tag sequence was situated at position 34–51 within the assay amplification region. The SP6 promotor was located at the end of the synthetic construct to allow for the synthesis of RNA transcripts.

Synthetic constructs (GenScript, Piscataway, NJ, USA) containing a 537 bp region of the nucleoprotein gene (Supplementary File S1) of a representative of each filovirus species (Table 1) were used to generate standard templates. In addition to the primer binding sites, each construct also contained the SP6 promoter sequence as well as a control tag sequence (position 229–247) that translated to amino acids FVQPCR (Figure 1, Supplementary File S1). This control tag would indicate contamination if found in a sequence obtained from a positive surveillance sample. Briefly, 10 ng of each construct was used to amplify filovirus inserts by utilizing the M13 priming sites on the vector. Inserts were in vitro transcribed from purified PCR products using the MEGAscript Kit (Ambion, Austin, TX, USA) according to the manufacturer’s instructions. RNA transcripts were digested with DNase (Ambion, Austin, TX, USA) twice before purification with the RNeasy MinElute Clean-up Kit (Qiagen, Hilden, Germany) according to the manufacturer’s instructions. RNA transcripts were tested with the SYBR Green qPCR (qPCR) to determine the absence of residual template DNA before synthesizing complementary DNA.

### 2.3. Reverse Transcription

Reverse transcription was performed on all samples using the following protocol. RNA (5 µL for samples or 1 µL standard RNA) was added to 100 ng random hexamers (Integrated DNA technologies, Coralville, IA, USA) and 1 µL dNTP mix (10 mM, Thermo Fisher Scientific, Waltham, MA, USA) in a final volume of 12 µL. This mixture was incubated at 65 °C for 5 min, followed by one-minute incubation on ice. Seven µL reaction mix containing 1× SSIV buffer (Invitrogen, Carlsbad, CA, USA), 5 M dithiothreitol (Invitrogen, Carlsbad, CA, USA), 40 U Ribolock RNase inhibitor (Thermo Fisher Scientific, Waltham, MA, USA), and 200 U SuperScript IV reverse transcriptase (Invitrogen, Carlsbad, CA, USA) was added and incubated at 23 °C for 10 min, 50 °C for 30 min, followed by incubation at 80 °C for 10 min. All reactions were stored at −70 °C until use.

### 2.4. SYBR Green qPCR

The primer concentration, incubation temperature and duration, ramp rate, and reaction volume were optimized. All qPCR reactions were performed using the FastStart Essential DNA Green Master Kit (Roche, Basel, Switzerland) and the QuantStudio 5 real-time PCR system (Applied Biosystems, Waltham, MA, USA). Optimized reactions were performed in a final volume of 20 µL containing 0.5 µM of each primer and 1× FastStart Essential DNA Green Master reaction mix. Reactions were incubated at 95 °C for 10 min, followed by 45 cycles of 95 °C for 20 s, 58 °C for 20 s, 72 °C for 20 s, and 79 °C for 10 s with the acquisition of fluorescence (ramp rate of 1.6 °C/s), followed by a melt curve stage at 95 °C for 15 s, 60 °C for 60 s, and 95 °C for 1 s (ramp rate of 0.15 °C/s). Analysis was performed using QuantStudio Design and Analysis software v1.5.2 (Applied Biosystems, Waltham, MA, USA) using the baseline threshold algorithm with multi-peak calling enabled.

### 2.5. Analytical Sensitivity

In vitro transcribed RNA was diluted in nuclease-free water to represent 10^0^–10^9^ copies/µL. For each construct, all dilutions were tested in triplicate, and probit regression analysis was performed using MedCalc Statistical software version 18.10.2 (MedCalc Software Ltd., Brussels, Belgium) to determine the limit of detection (LOD).

Titrated cultures of an EBOV isolate (SVPL 983/14, from an EBOV patient in Sierra Leone 2014, passage 4, 1.5 × 10^6^ TCID_50_/_mL_) and a MARV isolate (from the Watsa outbreak, Democratic Republic of Congo 2000, passage 6, 2.5 × 10^7^ TCID_50_/_mL_) were prepared in Vero C1008 (American Type Cell Collection [ATCC], Manassas, VA, USA) cell culture. The cell culture and virus culturing were performed using standard protocols in the biosafety level 4 containment laboratory at the NICD/NHLS. Ten-fold serial dilutions of the isolates were prepared in cell culture fluid. One hundred µL of each dilution was transferred to AVL extraction buffer (Qiagen, Hilden, Germany) and 560 µL of absolute ethanol was added. The inactivated samples were then removed from the containment laboratory for further manipulation under biosafety level 2 conditions. RNA was extracted with the Viral RNA Mini Kit (Qiagen, Hilden, Germany) according to the manufacturer’s instructions in an elution volume of 60 µL. The RNA was tested in triplicate with the SYBR Green assay, and for comparative purposes, once with a hydrolysis probe-based assay [30].

### 2.6. Hydrolysis Probe-Based qPCR

All reactions were performed using the OneStep RT-PCR Kit (Qiagen, Hilden, Germany) and the QuantStudio 5 real-time PCR system (Applied Biosystems, Waltham, MA, USA). Separate reactions were performed for EBOV and MARV serially diluted cell culture virus and each dilution was tested once. Reactions were performed in a final volume of 25 µL containing 2.5 mM OneStep RT-PCR buffer, 0.4 mM dNTP mix, 0.6 µM forward primer (Table 2), 0.7 µM reverse primer (Table 2), 0.1 µM probe (Table 2), 32 µg/mL bovine serum albumin, 2 µL OneStep RT-PCR enzyme, and 5 µL RNA. Reactions were incubated at 50 °C for 30 min, 95 °C for 15 min, followed by 45 cycles of 95 °C for 15 s, 52 °C for 25 s (with acquisition of fluorescence), and 72 °C for 20 s.

### 2.7. Spiked Sample Panel

To simulate the bat surveillance samples, pooled negative sera (sample number: SVPL 123/13) collected from a captive colony of *Rousettus aegyptiacus* bats [31] were spiked with serially diluted titrated EBOV and MARV (refer to Section 2.5) and tested in triplicate with the SYBR Green qPCR assay and for comparative purposes with a hydrolysis probe based qPCR assay [30].

### 2.8. Field Sample Panel

To assess the use of the assay in field applications, samples previously collected from Egyptian rousette bats (*R. aegyptiacus*) from Matlapitsi Cave, South Africa (GPS: −24.11487, 30.12151) during 2018–2021 were selected for testing (*n* = 990). The samples were collected and processed as described previously [32] and included sample types frequently used for non-destructive and non-invasive biosurveillance purposes (Table 3).

**Figure 2:**
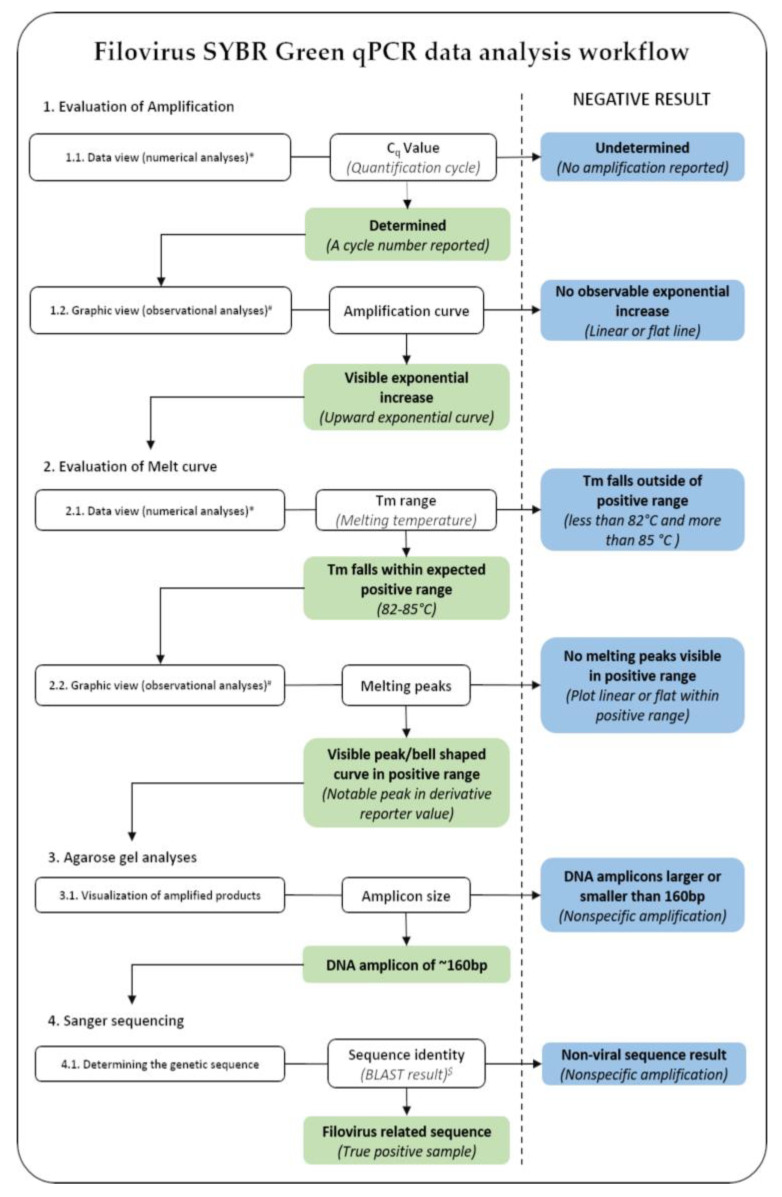
Filovirus SYBR Green qPCR data analysis workflow. The diagram depicts the workflow to assess individual samples at several steps to determine the additional analyses required for suspected positive samples. Results indicated in light blue represent negative samples and no further analyses were needed. Findings indicated in light green require further data analyses indicated with arrows. *: Numerical values of the C_q_/Tm provided; #: Visual representation of the amplification/melt curve plots; $: Analyses of nucleotide sequences using the BLAST function of the National Center for Biotechnology Information (NCBI) database (accessible online at https://blast.ncbi.nlm.nih.gov/Blast.cgi (accessed on 10 January 2023)).

Samples were tested in batches of either 48 or 96 sample formats using the methodology described in Section 2.3 and Section 2.4. All qPCR results were analyzed using the QuantStudio Design and Analysis software v1.5.2 (Applied Biosystems, Waltham, MA, USA) and visually inspected (Figure 2). In order to achieve the highest sensitivity possible, no quantification cycle (Cq) cutoff value was implemented and all samples with a Cq value was deemed potentially positive pending further analysis. The melting curves for samples with a Cq value and corresponding amplification curve were inspected for visible melting peaks. Samples with a melting temperature (Tm) value within the target range of 82–85 °C were deemed as potential positives. These samples were further analyzed on a 2% agarose gel in a horizontal submarine electrophoresis unit (Hoefer, Holliston, MA, USA) at 120 V until adequate separation of the GeneRuler 100 bp DNA Ladder (Thermo Fisher Scientific, Waltham, MA, USA). Any observable amplicons of approximately 160 bp were subsequently subjected to Sanger sequencing using the BigDye Terminator v3.1 Cycle Sequencing Kit (Thermo Fisher Scientific, Waltham, MA, USA) and the Filo Rev primer for initial confirmation. Larger and smaller amplicons were initially included for sequencing analyses to determine the source of any non-specific amplification.

## 3. Results

### 3.1. Analytical Sensitivity

#### 3.1.1. Standard Templates

**Figure 3:**
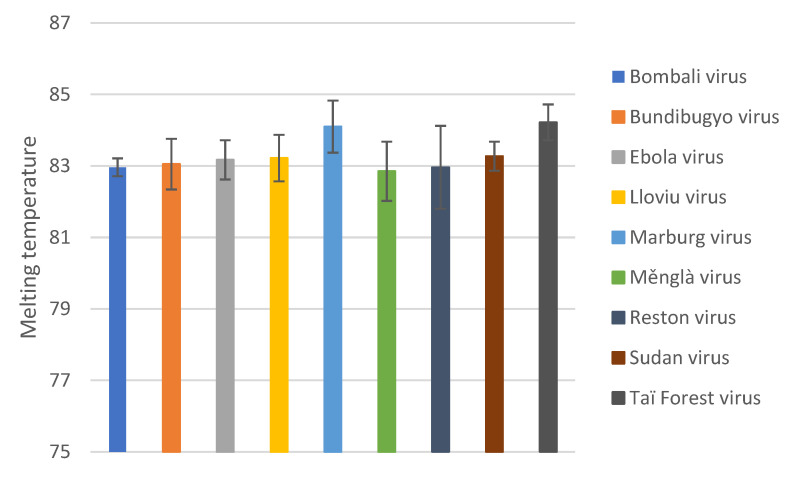
Average melting temperatures of the amplified filovirus standard templates (10^0^–10^9^ copies/reaction). Error bars indicate the standard deviation.

The optimized SYBR Green qPCR assay detected all standard templates tested (Table 4) with Cq values ranging from 10.23 to 43.59. For BOMV, BDBV, EBOV, MLAV, and SUDV, all dilutions in triplicate were detected. For MARV and REST, only two of the three replicates were detected at 10^0^ copies/reaction. Probit regression analysis could not be performed for only positive results, thus the LOD for these viruses are reported as the theoretical LOD of qPCR of three copies/reaction [33]. For TAFV only one of the three replicates at 10^0^ copies/reaction were detected with probit regression analysis indicating a LOD of 2.1 copies/reactions, reported as the theoretical LOD of qPCR of three copies/reaction. For LLOV only one of the three replicates were detected at 10^0^ copies/reaction and two of three replicates were detected at 10^1^ copies/reaction, with probit regression analysis indicating a LOD of 31.7 copies/reaction. The average Tm of the amplified standard templates were 82.85–84.1 °C (Figure 3), with primer dimers indicated with a Tm of 74–75 °C for no template controls.

**Figure 4:**
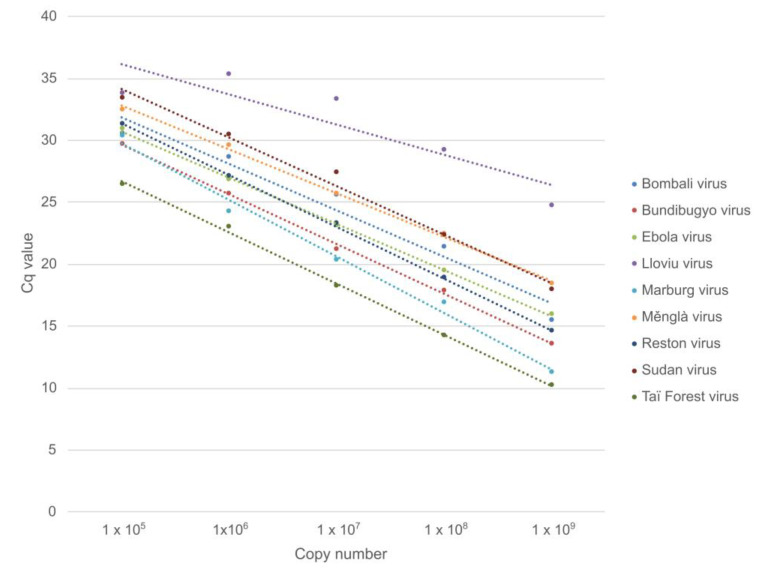
Standard curves for the serially diluted (10^5^–10^9^ copies/reaction) standard templates.

High variability was observed for low copy numbers (10^4^ copies/reaction and below), most likely due to primer dimer formation, as observed with melting curve analysis and gel electrophoresis. To minimize the influence of primer dimers on the linearity and assay characteristics, only results for 10^5^–10^9^ copies/reaction were included for the construction of standard curves (Figure 4). Standard curves yielded correlation coefficients (R^2^) of >0.95 for all standard templates except for LLOV (Table 5).

#### 3.1.2. Titrated Cell Culture Virus

Serially diluted cell culture viruses were tested in triplicate with the SYBR Green assay and for comparative purposes once with a hydrolysis probe-based assay [30].

**Figure 5:**
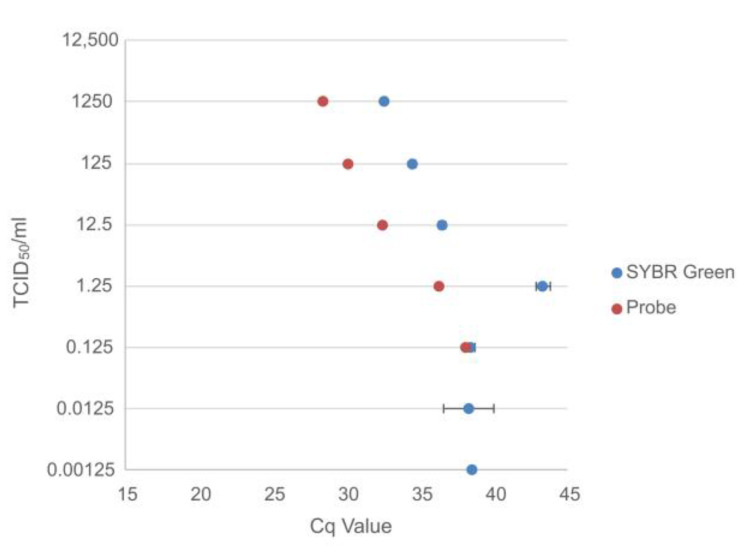
Comparison of the Cq values for the probe-based assay and the average Cq values for the SYBR Green assay for the titrated (TCID_50_/mL) serially diluted Ebola virus.

The probe-based assay detected EBOV up to a concentration of 0.125 TCID_50_/mL while the SYBR Green assay detected two out of the three replicates at 0.125 and 0.0125 TCID_50_/mL, and one out of the three replicates at 0.00125 TCID_50_/mL (Figure 5).

**Figure 6:**
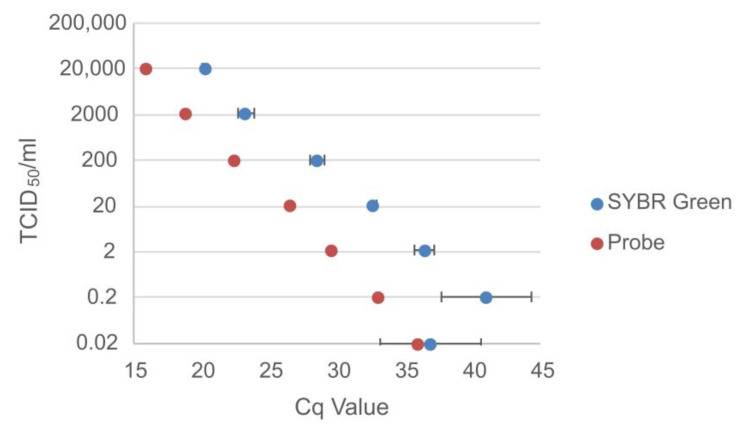
Comparison of the Cq values for the probe-based assay and the average Cq values for the SYBR Green assay for the titrated (TCID_50_/mL) serially diluted Marburg virus.

The probe-based assay detected MARV up to a concentration of 0.02 TCID_50_/mL while the SYBR Green assay detected two out of the three replicates up to a concentration of 0.02 TCID_50_/mL (Figure 6).

### 3.2. Spiked Sample Panel

To simulate bat surveillance samples, pooled negative bat sera were spiked with serially diluted titrated EBOV and MARV.

**Figure 7:**
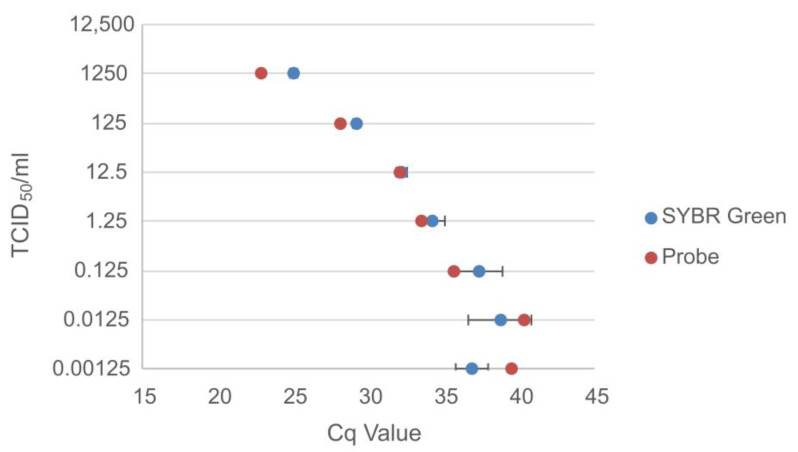
Comparison of the Cq values for the probe-based assay and the average Cq values for the SYBR Green assay for the negative bat sera spiked with the titrated (TCID_50_/mL) serially diluted Ebola virus.

The probe-based assay was able to detect EBOV up to a concentration of 0.00125 TCID_50_/mL, while the SYBR Green assay was able to detect two out of the three replicates at this concentration (Figure 7), resulting in a sensitivity of 95.2% (95% confidence interval: 74.13–99.8%).

**Figure 8:**
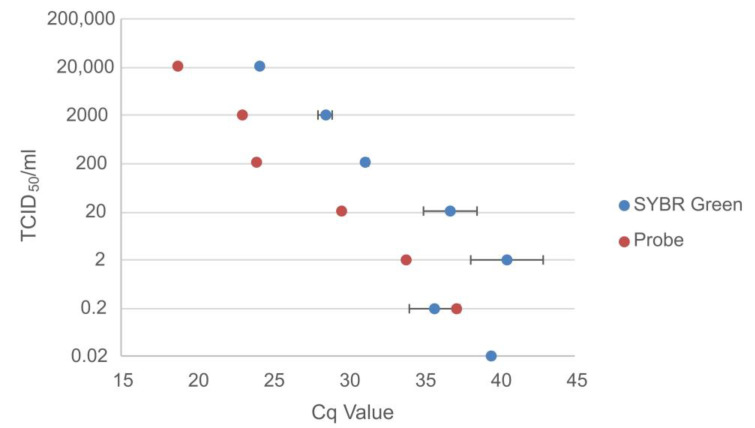
Comparison of the Cq values for the probe-based assay and the average Cq values for the SYBR Green assay for negative bat sera spiked with the titrated (TCID_50_/mL) serially diluted Margburg virus.

The probe-based assay was able to detect MARV up to a concentration of 0.2 TCID_50_/mL, while the SYBR Green assay was able to detect two out of the three replicates at a concentration of 0.02 TCID_50_/mL (Figure 8), resulting in a higher sensitivity for the SYBR Green assay compared to the probe-based assay for the detection of MARV.

### 3.3. Field Sample Panel

All samples included in the field sample testing were negative for any filovirus RNA. Overall, there was amplification not related to primer dimers (Tm ~73–78 °C) in 39 of the samples (3.9%) with a Tm in the target range of 82–85 °C. These samples were considered potential positives and required follow-up with agarose gel analyses (Table S2). Amplicons were either larger or smaller than the expected 160 bp target amplicon, indicating non-specific amplification and were deemed negative for filovirus RNA. Non-specific amplification was predominantly observed in the fecal samples (*n* = 32, 7.6%) and less frequently in oral swabs (*n* = 2, 3.1%) and urine (*n* = 5, 2.1%). No non-specific amplification was observed in the rectal swabs.

To determine the source of the non-specific amplification, selected bands were purified from agarose gels and sequenced and identified to be predominantly *Cellvibrio* sp., and to a lesser extent *Diaminobutyricimonas* sp.

## 4. Discussion

Filovirus hemorrhagic fever is considered emerging and in 2022, three outbreaks were reported from Uganda, the Democratic Republic of the Congo, and Ghana [34,35]. In recent decades, bats have been identified as reservoirs for numerous zoonoses of public and animal health concern [25]. Considering the increased interest in pathogen discovery in bats [21], it is not surprising that several novel filoviruses have been identified in bat species. These novel filoviruses were detected using degenerate nested consensus RT-PCR [11,12,18], in addition to genus and species-specific PCR assays [14] or with a two-well pan-filovirus RT-qPCR assay [16]. In addition to the assays above-mentioned, commercial assays are available but are specific for members of the *Ebolavirus* and *Marburgvirus* genera, with no assay having been evaluated against all mammalian filovirus species. Thus, surveillance samples should be tested with multiple assays to limit the possibility of false negative results. This approach would not be economically feasible, or in cases with limited sample material where it is not possible for surveillance studies targeting multiple viral families. To overcome these limitations, a two-step SYBR Green assay was developed. This would allow for a single cDNA preparation that can be used for multiple PCR assays targeting different viral families, maximizing the use of limited sample material. SYBR Green-based assays have the advantage that target-specific probes are not required for detection, resulting in lower costs, a more straightforward assay design, and the ability to be adapted to detect multiple pathogens.

The pan-filovirus SYBR Green assay described here detected representatives of all nine mammalian filovirus species known at the time of the study. Due to the diversity observed, a single primer pair containing multiple degenerate bases was used in this assay with an analytical sensitivity of 3–31.7 copies/reaction. At low copy numbers (10^4^ copies/reaction and below), the reactions were not linear; therefore, this assay should not be used for quantification. This nonlinearity was most likely caused by the formation of significant primer dimers due to the degenerate primer set. Although a reduction in the concentration of primers used in the assay reduced the formation of primer dimers, this also decreased the assay’s sensitivity. To minimize the influence of primer dimers on the Cq values, an additional step was included in the cycling conditions. The fluorescence signal was acquired at 79 °C, a temperature above the melting temperature of the primer dimers and below the melting temperature of the desired PCR products. Although these measures did not eliminate the primer dimer formation, they were readily distinguished from the desired products via melting curve analysis and should be used in combination with the amplification curves to determine the result. Overall, the assay performed at the same level as a probe-based assay for the detection of EBOV and MARV. Compared to the probe-based assay, the sensitivity of the SYBR Green assay was >95%. Using filovirus spiked bat sera as the template produced similar Cq values and melting temperatures as observed for the titrated cell culture virus, indicating this assay’s suitability for bat surveillance samples. A limitation of this study is that the specificity of this assay was not investigated with a panel of heterologous viruses. Based on the in silico sequence analysis of other viruses associated with bats or hemorrhagic fevers, non-specific amplification is unlikely. However, as degenerate primers are used in conjunction with a non-specific detection method, the possibility of non-specific amplification products with similar Tm values as the filovirus target cannot be ruled out. To assess if this assay would be applicable to surveillance studies, a panel of samples representing various sample types were tested. All samples were collected from *R*. *aegyptiacus*. In addition to the *Filoviridae*, evidence of members of at least 16 viral families have been detected in the Egyptian rousette bat including *Adeno*-, *Astro*-, *Corona*-, *Flavi*-, *Herpes*-, *Nairo*-, *Orthomyxo*-, *Orthoreo*-, *Papilloma*-, *Paramyxo*-, *Phenu*-, *Polyma*-, *Pox*-, *Reo*-, *Rhabdo*-, and *Togaviridae* [36]. A total of 990 samples, representing non-destructive and non-invasive sample types, were tested with the SYBR Green qPCR assay. The majority of samples (>96%) were determined to be negative based on the amplification and Tm data. Amplification was identified in 3.9% of samples with a Tm in the target range and required further analysis. Upon the analysis of amplicons using gel electrophoresis, these samples were determined to be of incorrect size with subsequent sequencing, indicating non-specific amplification of bacterial DNA. Although non-specific amplification could be resolved using gel electrophoresis, it is recommended that all potential positives be confirmed with DNA sequencing given the significant degeneracy of the primer set and non-specific detection method.

To summarize, viral discovery in bats has significantly increased with an analysis of the published studies indicating the need to optimize surveillance strategies for more efficient and targeted pathogen detection [25]. The developed pan-filovirus SYBR Green assay will allow for the economic and efficient detection of mammalian filoviruses in bat samples. Improved surveillance for filoviruses in bats will aid in our understanding of the role of bats in the natural ecology of the virus and the diversity of filoviruses. This, in turn, may serve to assist in being better prepared for future outbreaks of filovirus hemorrhagic fever.

## Figures and Tables

**Table 1:** Filovirus sequences representative of each mammalian filovirus species used for the synthetic constructs.

Virus	Species	Genus	Genbank Accession Number	Host	Country	Year
Bombali virus	*Bombali ebolavirus*	*Ebolavirus*	NC039345	*Mops condylurus*	Sierra Leone	2016
Bundibugyo virus	*Bundibugyo ebolavirus*	*Ebolavirus*	KC545396	*Homo sapiens*	Democratic Republic of the Congo	2012
Ebola virus	*Zaire ebolavirus*	*Ebolavirus*	MG572235	*Homo sapiens*	Democratic Republic of the Congo	1995
Lloviu virus	*Lloviu cuevavirus*	*Cuevavirus*	NC016144	*Miniopterus schrebersii*	Spain	2003
Marburg virus	*Marburg marburgvirus*	*Marburgvirus*	MG725616	*Rousettus aegyptiacus*	South Africa	2013
Měnglà virus	*Mengla dianlovirus*	*Dianlovirus*	KX371887	*Rousettus* sp.	China	2015
Reston virus	*Reston ebolavirus*	*Ebolavirus*	JX477165	Swine	Philippines	2009
Sudan virus	*Sudan ebolavirus*	*Ebolavirus*	KC545391	*Homo sapiens*	Uganda	2012
Täi Forest virus	*Tai Forest ebolavirus*	*Ebolavirus*	MH121167	*Homo sapiens*	Cote d’Ivoire	1994

**Table 2:** Primer and probes used for the hydrolysis probe-based assay targeting the RNA-dependent RNA polymerase gene of Ebola- and Marburgvirus [30].

Target	Primer/Probe	Sequence (5′-3′) ^1^	Position on Genome ^2^
Ebolavirus	FiloA2.4	AGCATTTCCTAGCAATATGATGGT	13340
	Filo B	TGTGGTGGGTTATAATAATCACTGACATG	13603
	FAMEBOg	FAM-CCAAAATCATCACTIGTGTGGTGCCA-BHQ1	13411
Marburgvirus	FiloA2.3	AAGCATTCCCTAGCAACATGATGGT	13249
	Filo B-Ra	GTGAGGAGGGCTATAAAAGTCACTGACATG	13512
	FAMMBG	FAM-CCTATGCTTGCTGAATTGTGGTGCCA-BHQ1	13320

^1^ FAM: 6-carboxyfluorescein, BHQ1: Black Hole Quencher 1. ^2^ Nucleotide positions are numbered according to NC002549 (Ebolavirus) and NC001608 (Marburgvirus).

**Table 3:** Number and sample types included to assess the SYBR Green assay performance on the field collected samples obtained from the Egyptian rousette bat.

Sample Type	Count	Approach
Urine ^1^	410	Non-invasive
Fecal ^2^	422	
Oral swab	65	Non-destructive
Rectal swab	93	

^1^ Population level urine samples collected on plastic trays underneath roosting bats. ^2^ Population level fecal samples collected from the cave floor underneath roosting bats.

**Table 4:** The average SYBR Green Cq values (standard deviation, SD) for serially diluted standard templates tested in triplicate.

Copy Number	Bombali Virus	Bundibugyo Virus	Ebola Virus	Lloviu Virus	Marburg Virus	Měnglà Virus	Reston Virus	Sudan Virus	Taï Forest Virus
10^−1^	Neg	Neg	Neg	Neg	Neg	Neg	Neg	Neg	Neg
10^0^	33.62 (0.36)	39.29 (0.99)	39.23 (0.75)	40.17 ^1^	41.98 (1.52) ^2^	37.55 (1.32)	41.98 (1.52) ^2^	31.30 (0.40)	43.59 ^1^
10^1^	33.26 (0.56)	35.12 (1.77)	35.55 (1.44)	36.59 (3.25)^2^	39.35 (0.89)	39.69 (1.28)	37.54 (0.89)	38.71 (3.92)	37.84 (1.61)
10^2^	33.11 (1.02)	39.98 (1.01)	37.31 (0.51)	31.22 (5.68)	40.53 (4.83)	38.93 (1.95)	32.67 (4.83)	31.92 (0.38)	34.86 (0.50)
10^3^	35.06 (1.27)	37.63 (0.32)	35.59 (0.92)	36.35 (2.58)	34.46 (0.13)	36.07 (1.68)	40.54 (1.83)	42.48 (0.53)	34.34 (0.29)
10^4^	32.42 (0.26)	33.92 (0.35)	36.17 (1.41)	35.53 (1.48)	34.09 (0.37)	38.18 (1.63)	35.56 (0.08)	37.29 (2.01)	30.38 (0.22)
10^5^	30.62 (0.37)	29.76 (0.28)	30.98 (0.41)	33.77 (3.22)	30.39 (0.27)	32.51 (1.1)	31.34 (0.12)	33.46 (0.46)	26.42 (0.09)
10^6^	28.65 (0.23)	25.65 (0.18)	26.84 (0.17)	35.29 (0.04)	24.29 (0.34)	29.59 (0.13)	27.13 (0.14)	30.51 (0.35)	23.02 (0.15)
10^7^	25.62 (0.25)	21.18 (0.50)	23.09 (0.50)	33.36 (0.41)	20.40 (0.51)	25.68 (0.14)	23.31 (0.12)	27.42 (0.82)	18.24 (0.18)
10^8^	21.40 (0.24)	17.84 (0.18)	19.49 (0.47)	29.21 (0.40)	16.92 (0.43)	22.47 (0.31)	18.88 (0.04)	22.37 (0.74)	14.22 (0.26)
10^9^	15.50 (0.21)	13.61 (0.47)	16.01 (0.31)	24.73 (0.80)	11.26 (0.04)	18.45 (0.24)	14.63 (0.06)	17.98 (0.74)	10.23 (0.16)

^1^ Only 1/3 replicates were positive. standard deviation could not be determined. ^2^ Only 2/3 replicates were positive.

**Table 5:** The SYBR Green assay characteristics for serially diluted standard templates.

Virus	Limit of Detection (Copies/Reaction)	Correlation Coefficient (R^2^)
Bombali virus	3	0.960
Bundibugyo virus	3	0.998
Ebola virus	3	0.998
Lloviu virus	31.7	0.791
Marburg virus	3	0.990
Měnglà virus	3	0.998
Reston virus	3	0.999
Sudan virus	3	0.987
Taï Forest virus	3	0.998

## Data Availability

The data are contained within the article or supplementary materials.

## Supplementary Materials

The following supporting information can be downloaded at: https://www.mdpi.com/article/10.3390/v15040987/s1, Table S1: Publically available sequences used for primer design; Supplementary File S1: Synthetic constructs sequences, Table S2: SYBR Green qPCR results for field samples.
