# Supplementary material for: Development of a Pan-Filoviridae SYBR Green qPCR Assay for Biosurveillance Studies in Bats
Source: Viruses. 2023 Apr 17;15(4):987. doi: 10.3390/v15040987 (PMC10145118; doi:10.3390/v15040987)
Supplement: Supplementary file 1 [file viruses-15-00987-s001.zip › Supplementary file 1_Synthetic constructs sequences.pdf]

```
                       10        20        30        40        50        60
              ....|....|....|....|....|....|....|....|....|....|....|....|
BOMV_NC_039345.1    ATGCCAACGATGCTGTTATCGCTAATTCAGTTGCTCAGGCACGCTTTTCAGGCTTGTTGA
BDBV_KC545396       ATGCTAATGATGCCGTCATTGCCAACTCTGTAGCTCAAGCTCGTTTCTCCGGATTGTTGA
LLOV_NC_016144      ATGCTAATGATGCAATCATTGCCAATTCAATATCCCAAACACGATTTTCAGGATTGTTAA
MARV_MG725616       ATGCCTATGACAGTATCATCAGTAATTCAGTAGGTCAAACTAGATTCTCAGGACTTCTTA
MLAV_KX371887       ATGCCTATGACAATGTCATCAGCAATTCCATAAACCAAACACGCTTCTCAGGACTGCTGA
RESTV_JX477165      ATGCCAATGATGCTGTCATTGCTAATTCAGTTGCTCAGGCTCGCTTTTCAGGACTCCTAA
SUDV_KC545391       ATGCGAACGACACAGTAATATCTAATTCTGTTGCCCAAGCAAGGTTCTCTGGTCTTCTGA
TAFV_MH121167       ATGCTAACGATGCTGTCATCGCAAACTCTGTAGCTCAAGCACGTTTTTCAGGATTATTGA
EBOV_MG572235       ATGCCAACGACGCTGTGATTTCAAATTCAGTGGCTCAAGCTCGTTTTTCAGGTTTATTGA
Filo Forward        ------------------------------------------------------------
Filo Reverse        ------------------------------------------------------------
Control tag_FVQPCR  ------------------------------------------------------------
SP6_complement      ------------------------------------------------------------

                       70        80        90       100       110       120
              ....|....|....|....|....|....|....|....|....|....|....|....|
BOMV_NC_039345.1    TCGTCAAGACAGTGCTGGACCACATTCTGCAGAAAACAGACCGTGGAGTCCGTCTTCATC
BDBV_KC545396       TAGTCAAAACAGTGCTTGATCATATCCTCCAAAAAACAGAGCACGGAGTTCGCCTGCATC
LLOV_NC_016144      TCGTGAAGACTGTCCTAGAGCATATTCTCCAAAAAACTGAGGCAGGGGTCCAACTTCATC
MARV_MG725616       TTGTGAAAACAGTTCTCGAGTTCATCTTGCAAAAAACTGATTCAGGGGTGACACTACATC
MLAV_KX371887       TTGTAAAGACTGTCTTGGAACATATCCTCCAAAGGACTGAAAATGGAGTCATTCTTCACC
RESTV_JX477165      TTGTCAAAACCGTTCTTGACCATATTCTGCAAAAAACCGACCAAGGAGTAAGACTTCACC
SUDV_KC545391       TTGTAAAGACTGTTCTGGACCACATCCTACAAAAAACCGATCTTGGAGTACGACTTCATC
TAFV_MH121167       TCGTTAAAACAGTGCTAGATCACATCCTTCAGAAAACAGAGCACGGAGTGCGTCTTCATC
EBOV_MG572235       TTGTCAAAACAGTACTTGATCATATCCTACAAAAGACAGAACGCGGAGTTCGTCTCCATC
Filo Forward        ------------------------------------------------------------
Filo Reverse        ------------------------------------------------------------
Control tag_FVQPCR  ------------------------------------------------------------
SP6_complement      ------------------------------------------------------------

                      130       140       150       160       170       180
              ....|....|....|....|....|....|....|....|....|....|....|....|
BOMV_NC_039345.1    CTTTAGCACGTACAGCCAAGGTCAGGAACGAGGTCCATTCGTTCAAAGCTGCTCTTAACT
BDBV_KC545396       CCTTGGCGCGAACAGCCAAAGTCAAAAATGAGGTAAGCTCTTTTAAGGCCGCTTTAGCCT
LLOV_NC_016144      CATTAGCTCGAACCTCGAAAGTCAAAGGAGAACTCCTTGCATTCAAATCAGCACTGGAAG
MARV_MG725616       CTTTGGTGCGGACCTCCAAAGTAAAAAATGAAGTTGCTAGTTTCAAGCAGGCGTTGAGCA
MLAV_KX371887       CTCTTGTCAGAACCTCCAAGGTGAAGAGTGAAGTTGAGAGCTTCAAAGTTGCATTGAGAG
RESTV_JX477165      CTTTGGCCCGAACAGCCAAAGTGCGTAATGAGGTTAATGCATTTAAGGCCGCCCTAAGCT
SUDV_KC545391       CACTGGCCAGGACAGCAAAAGTCAAGAATGAGGTCAGTTCATTCAAGGCAGCTCTTGGCT
TAFV_MH121167       CTTTGGCAAGAACTGCTAAGGTCAAGAACGAAGTAAATTCCTTTAAGGCTGCCCTTAGCT
EBOV_MG572235       CTCTTGCAAGGACCGCCAAGGTAAAAAATGAGGTGAACTCCTTTAAGGCTGCACTCAGCT
Filo Forward        ------------------------------------------------------------
Filo Reverse        ------------------------------------------------------------
Control tag_FVQPCR  ------------------------------------------------------------
SP6_complement      ------------------------------------------------------------

                      190       200       210       220       230       240
              ....|....|....|....|....|....|....|....|....|....|....|....|
BOMV_NC_039345.1    CCCTGGCTCAGCATGGGGAATATGCCCCCTTTGCTCGACTTTTAAACCttcgttcaacct
BDBV_KC545396       CACTAGCACAACATGGAGAATATGCCCCGTTCGCTCGTCTGCTGAATCttcgttcaacct
LLOV_NC_016144      CGTTGGCCTCACACAGAGAGTATGCACCATTTGCACGGCTCTTGAACCttcgttcaacct
MARV_MG725616       ACCTAGCCCGACATGGAGAATACGCACCATTTGCACGGGTTCTGAATTttcgttcaacct
MLAV_KX371887       GTCTGGCAAGACACAAAGAATACGCACCTTTTGCCCGGGTATTGAACTttcgttcaacct
RESTV_JX477165      CACTTGCTAAGCACGGGGAATATGCCCCTTTTGCTCGCCTTCTCAATCttcgttcaacct
SUDV_KC545391       CACTTGCCAAGCATGGAGAATATGCTCCATTTGCACGTCTCCTCAATCttcgttcaacct
TAFV_MH121167       CGCTAGCACAACATGGAGAGTATGCTCCTTTTGCTCGCTTGCTGAATCttcgttcaacct
EBOV_MG572235       CCCTGGCCAAGCATGGAGAGTATGCTCCTTTCGCCCGACTTTTGAACCttcgttcaacct
Filo Forward        ---------------GRGARTAYGCNCCNTTYGC--------------------------
Filo Reverse        ------------------------------------------------------------
Control tag_FVQPCR  ------------------------------------------------pttcgttcaacct
```

```
SP6_complement       ------------------------------------------------------------

                             250       260       270       280       290       300
                     ....|....|....|....|....|....|....|....|....|....|....|....|
BOMV_NC_039345.1     tgtcgtTCGAACATGGGCTGTATCCTCAACTCTCTGCAATTGCACTTGGTGTGGCTACTG
BDBV_KC545396        tgtcgtTTGAGCATGGGCTTTTCCCTCAACTTTCTGCAATTGCTTTGGGAGTAGCAACTG
LLOV_NC_016144       tgtcgtTAGAACATGGCCTGTATCCTCAGCTCTCTGCCATCGCGCTTGGAGTTGCTACAG
MARV_MG725616        tgtcgtTCGAGCATGGACTCTATCCTCAGCTCTCAGCAATTGCGCTGGGTGTGGCTACAG
MLAV_KX371887        tgtcgtTTGAGCATGGACTTTTCCCTCAACTCTCAGCAATTGCAATCGGGGTTGCAACAG
RESTV_JX477165       tgtcgtTTGAACATGGTCTCTACCCACAGTTATCAGCAATTGCTCTTGGAGTTGCCACAG
SUDV_KC545391        tgtcgtTGGAACATGGGCTTTATCCACAACTCTCAGCCATTGCTTTGGGTGTTGCAACTG
TAFV_MH121167        tgtcgtTCGAGCACGGACTGTTTCCTCAGCTTTCTGCAATTGCCCTAGGTGTCGCAACGG
EBOV_MG572235        tgtcgtTTGAGCATGGTCTTTTCCCTCAACTATCAGCAATTGCACTCGGAGTCGCCACAG
Filo Forward         ------------------------------------------------------------
Filo Reverse         ------------------------------------------------------------
Control tag_FVQPCR   tgtcgt
SP6_complement       ------------------------------------------------------------

                             310       320       330       340       350       360
                     ....|....|....|....|....|....|....|....|....|....|....|....|
BOMV_NC_039345.1     CACACGGCAGCACTTTGGCAGGGGTGAATGTTGGTGAGCAGTACCAACAGCTCAGGGAGG
BDBV_KC545396        CACATGGGAGCACTCTGGCAGGAGTCAATGTAGGAGAGCAATACCAACAACTGCGAGAAG
LLOV_NC_016144       CTCATGGTAGCACACTAGCAGGAGTTAATGTGAGTGAACAATACCAGCAACTCCGAGAGG
MARV_MG725616        CACACGGCAGCACATTGGCTGGTGTCAATGTCGGCGAACAATATCAACAGCTACGAGAGG
MLAV_KX371887        CACATGGAAGTACATTGGCTGGGGTGAGTGTTGGAGAGCAGTATCAACAACTACGGGAAG
RESTV_JX477165       CACATGGTAGCACCCTTGCAGGAGTTAATGTTGGTGAGCAGTATCAGCAGCTCAGAGAGG
SUDV_KC545391        CCCACGGGAGCACGCTGGCTGGTGTTAATGTAGGGGAGCAATATCAGCAACTGCGTGAGG
TAFV_MH121167        CACACGGCAGTACCCTGGCAGGAGTAAATGTGGGGGAACAGTATCAGCAACTACGAGAAG
EBOV_MG572235        CACACGGGAGTACCCTCGCAGGAGTAAATGTTGGAGAACAGTATCAACAACTCAGAGAGG
Filo Forward         ------------------------------------------------------------
Filo Reverse         --------------------------------ggngarcartaycarcarct
Control tag_FVQPCR
SP6_complement       ------------------------------------------------------------

                             370       380       390       400       410       420
                     ....|....|....|....|....|....|....|....|....|....|....|....|
BOMV_NC_039345.1     CTGCCACGGAGGCTGAGAAGCAGCTGCAGCAATATGCTGAAACACGTGAGCTGGACCACT
BDBV_KC545396        CAGCCACTGAGGCCGAAAAGCAGTTGCAGAAATATGCTGAATCTCGTGAACTTGATCACC
LLOV_NC_016144       CTGCAACAGAGGCTGAGAAACAATTACAACAGCACTCAGAGATGCGGGAATTGGAGACCC
MARV_MG725616        CTGCACATGATGCAGAGGTAAAATTACAAAGGCGACATGAACATCAGGAAATTCAAGCTA
MLAV_KX371887        CAGCCCATGATGCAGAACTTAAACTCCAGAGGCGCCGTGAACAAATGGAAATTTCATCAC
RESTV_JX477165       CTGCCACTGAAGCTGAGAAGCAACTCCAACAATATGCTGAGTCCAGAGAACTCGACAGCC
SUDV_KC545391        CTGCTACTGAAGCTGAAAAGCAACTCCAACAATATGCTGAGACACGTGAGTTGGACAACC
TAFV_MH121167        CAGCCACTGAGGCAGAAAAACAATTGCAGAAATACGCTGAATCTCGCGAGCTTGACCATC
EBOV_MG572235        CTGCCACTGAGGCTGAGAAGCAACTCCAACAATACGCAGAGTCTCGCGAACTTGACCATC
Filo Forward         ------------------------------------------------------------
Filo Reverse
Control tag_FVQPCR
SP6_complement       ------------------------------------------------------------

                             430       440       450       460       470       480
                     ....|....|....|....|....|....|....|....|....|....|....|....|
BOMV_NC_039345.1     TAGGCCTAGACACAAAAGAAAAGGAAATATTAATGAATTTCCATCAACGGAAAAATGAGA
BDBV_KC545396        TAGGTCTTGATGATCAGGAAAAGAAAATCCTAAAAGACTTCCATCAGAAAAAGAATGAGA
LLOV_NC_016144       TTGGACTGGATGAACAAGAAAGGAAGATCCTTGCAACATTCCACTCCCGGAAAAATGAGA
MARV_MG725616        TTGCCGAAGATGACGAGGAAAGGAAGATATTAGAACAGTTCCACCTTCAAAAAACTGAAA
MLAV_KX371887        TCGAACTTGACCTTGAAGAACAGAAAATTCTGGAACAATTCCATCAGCAGAAAACAGAAA
RESTV_JX477165       TAGGCCTAGACGATCAGGAAAGAAGAATACTAATGAACTTCCATCAGAAGAAAAATGAAA
SUDV_KC545391        TTGGGCTTGATGAGCAGGAAAAGAAGATTCTCATGAGCTTCCACCAGAAGAAGAATGAGA
TAFV_MH121167        TAGGTCTCGATGATCAAGAGAAGAAGATCTTGAAAGACTTCCATCAGAAGAAAAATGAAA
EBOV_MG572235        TTGGACTTGATGATCAGGAAAAGAAAATTCTTATGAACTTCCATCAGAAAAAGAACGAAA
Filo Forward         ------------------------------------------------------------
```

```
Filo Reverse
Control tag_FVQPCR
SP6_complement      ------------------------------------------------------------

                         490       500       510       520       530       540
                    ....|....|....|....|....|....|....|....|....|....|....|....|
BOMV_NC_039345.1    TTAGTTTCCAGCAAACAAATGCAATGGTGTCCCTTCGCAAGGAGAGACTAGCAAAACcgc
BDBV_KC545396       TCAGCTTCCAGCAGACGACAGCCATGGTCACACTGCGGAAAGAGAGATTGGCCAAATcgc
LLOV_NC_016144      TCAACATTCAACAGACCAGCTCGATATTAGCAATTCGTAAGGAACGACTCCGCAAGCcgc
MARV_MG725616       TCACACACAGTCAGACACTTGCCGTCCTCAGCCAGAAACGAGAAAAATTAGCTCGTCcgc
MLAV_KX371887       TAACTCACACACAGACTCTTGCTGTTCTAACACAGAAGAAAGAAAAGTTAGCCAAACcgc
RESTV_JX477165      TTAGTTTCCAGCAGACCAATGCAATGGTCACCCTTAGGAAAGAGCGACTGGCTAAATcgc
SUDV_KC545391       TCAGCTTCCAGCAAACTAACGCAATGGTAACCTTGAGGAAAGAGCGGCTGGCCAAACcgc
TAFV_MH121167       TCAGCTTCCAGCAGACAACAGCCATGGTCACACTACGGAAGGAAAGGCTAGCCAAGCcgc
EBOV_MG572235       TCAGCTTCCAGCAAACAAACGCTATGGTAACTCTAAGAAAAGAGCGCCTGGCCAAGCcgc
Filo Forward        ---------------------------------------------------------
Filo Reverse
Control tag_FVQPCR
SP6_complement      ---------------------------------------------------------cgc

                         550       560       570
                    ....|....|....|....|....|....|.
BOMV_NC_039345.1    ttctatagtgtcacctaaatagaataactct
BDBV_KC545396       ttctatagtgtcacctaaatagaataactct
LLOV_NC_016144      ttctatagtgtcacctaaatagaataactct
MARV_MG725616       ttctatagtgtcacctaaatagaataactct
MLAV_KX371887       ttctatagtgtcacctaaatagaataactct
RESTV_JX477165      ttctatagtgtcacctaaatagaataactct
SUDV_KC545391       ttctatagtgtcacctaaatagaataactct
TAFV_MH121167       ttctatagtgtcacctaaatagaataactct
EBOV_MG572235       ttctatagtgtcacctaaatagaataactct
Filo Forward
Filo Reverse
Control tag_FVQPCR
SP6_complement      ttctatagtgtcacctaaat
```
