# Supplementary material for: Development of a Pan-Filoviridae SYBR Green qPCR Assay for Biosurveillance Studies in Bats
Source: Viruses. 2023 Apr 17;15(4):987. doi: 10.3390/v15040987 (PMC10145118; doi:10.3390/v15040987)
Supplement: Supplementary file 1 [file viruses-15-00987-s001.zip › Table S1_Publically available sequences used for primer design.pdf]

Table S1. Publically available sequences used for primer design.

Genbank accession number	Virus	Host	Year	Country
KU182911	Bundibugyo virus	*Homo sapiens*	2007	Uganda
KR063673	Bundibugyo virus	*Homo sapiens*	2007	Uganda
NC_014373	Bundibugyo virus	*Homo sapiens*	2007	Uganda
MK028835	Bundibugyo virus	*Homo sapiens*	2007	Uganda
MK028834	Bundibugyo virus	*Homo sapiens*	2007	Uganda
KC545396	Bundibugyo virus	*Homo sapiens*	2012	Democratic Republic of Congo
KC545395	Bundibugyo virus	*Homo sapiens*	2012	Democratic Republic of Congo
KC545394	Bundibugyo virus	*Homo sapiens*	2012	Democratic Republic of Congo
KC545393	Bundibugyo virus	*Homo sapiens*	2012	Democratic Republic of Congo
MK028856	Bundibugyo virus	*Homo sapiens*	2007	Uganda
NC_039345	Bombali virus	*Mops condylurus*	2016	Sierra Leone
MT929366	Bombali virus	*Mops pumilus*	2018	Sierra Leone
MT929365	Bombali virus	*Mops condylurus*	2018	Guinea
MW056493	Bombali virus	*Mops condylurus*	2019	Kenya
MW056492	Bombali virus	*Mops condylurus*	2019	Kenya
MK340750	Bombali virus	*Mops condylurus*	2018	Kenya
MF319186	Bombali virus	*Chaerephon pumilus*	2016	Sierra Leone
MF319185	Bombali virus	*Mops condylurus*	2016	Sierra Leone
KR867676	Ebola virus	*Homo sapiens*	1995	Democratic Republic of Congo
AF086833	Ebola virus	*Homo sapiens*	1976	Democratic Republic of Congo
KR063671	Ebola virus	*Homo sapiens*	1976	Democratic Republic of Congo
KC242801	Ebola virus	*Homo sapiens*	1976	Democratic Republic of Congo
KC242791	Ebola virus	*Homo sapiens*	1977	Democratic Republic of Congo
KJ660348	Ebola virus	*Homo sapiens*	2014	Guinea
KJ660347	Ebola virus	*Homo sapiens*	2014	Guinea
KJ660346	Ebola virus	*Homo sapiens*	2014	Guinea
MH121168	Ebola virus	*Homo sapiens*	1976	Democratic Republic of Congo
MH121161	Ebola virus	*Homo sapiens*	2014	Sierra Leone
MG572234	Ebola virus	*Homo sapiens*	2014	Guinea
MK731988	Ebola virus	*Homo sapiens*	2018	Democratic Republic of Congo
MK163674	Ebola virus	*Homo sapiens*	2018	Democratic Republic of Congo
KP728283	Ebola virus	*Homo sapiens*	2014	Switzerland
KY426728	Ebola virus	*Homo sapiens*	2015	Sierra Leone
KM519951	Ebola virus	*Homo sapiens*	2014	Democratic Republic of Congo
KR006960	Ebola virus	*Homo sapiens*	2015	Liberia
KR006957	Ebola virus	*Homo sapiens*	2014	Liberia
KR006940	Ebola virus	*Homo sapiens*	2014	Liberia
KP271020	Ebola virus	*Homo sapiens*	2014	Democratic Republic of Congo
KP271019	Ebola virus	*Homo sapiens*	2014	Democratic Republic of Congo
KR006955	Ebola virus	*Homo sapiens*	2014	Liberia
MH613311	Ebola virus	*Homo sapiens*	2017	Democratic Republic of Congo
KT013256	Ebola virus	*Homo sapiens*	2014	Guinea
KT013254	Ebola virus	*Homo sapiens*	2014	Guinea
MK731994	Ebola virus	*Homo sapiens*	2018	Democratic Republic of Congo
MK731992	Ebola virus	*Homo sapiens*	2018	Democratic Republic of Congo
MG948591	Ebola virus	*Homo sapiens*	2014	Sierra Leone
MK163649	Ebola virus	*Homo sapiens*	2018	Democratic Republic of Congo
KY786009	Ebola virus	*Macaca fascicularis*	2001	Gabon
MH470382	Ebola virus	*Homo sapiens*	2015	Guinea
KT357859	Ebola virus	*Homo sapiens*	2015	Sierra Leone

KY805812	Ebola virus	*Homo sapiens*	2014	Sierra Leone
KY401672	Ebola virus	*Homo sapiens*	2014	Guinea
KY401665	Ebola virus	*Homo sapiens*	2014	Sierra Leone
KY744597	Ebola virus	*Homo sapiens*	2015	Liberia
KY471124	Ebola virus	*Homo sapiens*	2001	Gabon
KX013101	Ebola virus	*Homo sapiens*	2014	Nigeria
KX013099	Ebola virus	*Homo sapiens*	2014	Nigeria
KX013098	Ebola virus	*Homo sapiens*	2014	Nigeria
KX013095	Ebola virus	*Homo sapiens*	2014	Nigeria
KU220284	Ebola virus	*Homo sapiens*	2015	Liberia
KU052670	Ebola virus	*Homo sapiens*	2015	United Kingdom
MZ541881	Lloviu virus	*Miniopterus schreibersii*	2019	Hungary
MW775011	Lloviu virus	*Miniopterus schreibersii*	2019	Hungary
MW775010	Lloviu virus	*Nycteribia schmidlii*	2019	Hungary
NC_016144	Lloviu virus	*Miniopterus schreibersii*	2003	Spain
JF828358	Lloviu virus	*Miniopterus schreibersii*	2003	Spain
AY358025	Marburg virus	*Homo sapiens*	1975	South Africa
DQ217792	Marburg virus	*Homo sapiens*	1980	Kenya
KP985768	Marburg virus	*Homo sapiens*	2014	Uganda
KY047763	Marburg virus	*Homo sapiens*	2005	Angola
MG725616	Marburg virus	*Rousettus aegyptiacus*	2013	South Africa
JX458826	Marburg virus	*Homo sapiens*	1999	Democratic Republic of Congo
JX458850	Marburg virus	*Homo sapiens*	2000	Democratic Republic of Congo
DQ447651	Marburg virus	*Homo sapiens*	1999	Democratic Republic of Congo
JX458833	Marburg virus	*Homo sapiens*	1999	Democratic Republic of Congo
JX458858	Marburg virus	*Rousettus aegyptiacus*	2009	Uganda
JX458853	Marburg virus	*Rousettus aegyptiacus*	2008	Uganda
KC545388	Marburg virus	*Homo sapiens*	2012	Uganda
JX458856	Marburg virus	*Rousettus aegyptiacus*	2009	Uganda
JX458852	Marburg virus	*Rousettus aegyptiacus*	2008	Uganda
JX458854	Marburg virus	*Rousettus aegyptiacus*	2009	Uganda
JX458855	Marburgvirus	*Rousettus aegyptiacus*	2009	Uganda
FJ743676	Marburg virus	*Rousettus aegyptiacus*	2008	Uganda
FJ750957	Marburg virus	*Homo sapiens*	2007	Uganda
FJ743675	Marburg virus	*Hipposideros caffer*	2007	Uganda
FJ743671	Marburg virus	*Rousettus aegyptiacus*	2007	Uganda
KU059750	Marburg virus	*Homo sapiens*	2007	Uganda
FJ750958	Marburg virus	*Rousettus aegyptiacus*	2007	Uganda
FJ750956	Marburg virus	*Rousettus aegyptiacus*	2008	Uganda
FJ750955	Marburg virus	*Rousettus aegyptiacus*	2007	Uganda
FJ750954	Marburg virus	*Rousettus aegyptiacus*	2007	Uganda
FJ750953	Marburg virus	*Homo sapiens*	2007	Uganda
EU068110	Marburg virus	*Rousettus aegyptiacus*	N/A	Gabon
FJ743677	Marburg virus	*Rousettus aegyptiacus*	2008	Uganda
KX371887	Měnglà virus	*Rousettus aegyptiacus*	2015	China
KU179482	Ravn virus	*Homo sapiens*	1987	Kenya
JX458857	Ravn virus	*Rousettus aegyptiacus*	2009	Uganda
KY798012	Reston virus	*Sus scrofa*	2009	Philippines
KY798011	Reston virus	*Sus scrofa*	2008	Philippines
KY798010	Reston virus	*Sus scrofa*	2008	Philippines
KY798007	Reston virus	*Macaca fascicularis*	1992	Italy
KY798005	Reston virus	*Macaca fascicularis*	1989	United States of America
KY798006	Reston virus	*Homo sapiens*	1989	United States of America
FJ621585	Reston virus	*Sus scrofa*	2008	Philippines
KY008770	Reston virus	*Macaca fascicularis*	1989	United States of America

KY798009	Reston virus	*Macaca fascicularis*	1996	United States of America
MF540571	Reston virus	*Macaca fascicularis*	2015	Philippines
JX477166	Reston virus	*Macaca fascicularis*	1996	United States of America
JX477165	Reston virus	*Sus scrofa*	2009	Philippines
KR063670	Sudan virus	*Homo sapiens*	2000	Uganda
JN638998	Sudan virus	*Homo sapiens*	2011	Uganda
MH121169	Sudan virus	*Homo sapiens*	2004	Sudan
MH121163	Sudan virus	*Homo sapiens*	2000	Uganda
AY729654	Sudan virus	*Homo sapiens*	2000	Uganda
KR063670	Sudan virus	*Homo sapiens*	2000	Uganda
KC545392	Sudan virus	*Homo sapiens*	2012	Uganda
KC545390	Sudan virus	*Homo sapiens*	2012	Uganda
KC545391	Sudan virus	*Homo sapiens*	2012	Uganda
KC242783	Sudan virus	*Homo sapiens*	1979	Sudan
EU338380	Sudan virus	*Homo sapiens*	2004	Sudan
NC_006432	Sudan virus	*Homo sapiens*	2000	Uganda
FJ217162	Taï Forest virus	*Homo sapiens*	1994	Cote d'Ivoire
KP233864	Bat related, unclassified	*Rousettus leschenaulti*	2013	China
